# Verification of molecular subtyping of bladder cancer in the GUSTO clinical trial

**DOI:** 10.1002/2056-4538.12363

**Published:** 2024-02-01

**Authors:** Jon Griffin, Jenny Down, Lewis A Quayle, Paul R Heath, Ewan A Gibb, Elai Davicioni, Yang Liu, Xin Zhao, Jayne Swain, Dennis Wang, Syed Hussain, Simon Crabb, James WF Catto, the GUSTO Trial Management Group

**Affiliations:** ^1^ Department of Histopathology Sheffield Teaching Hospitals NHS Foundation Trust Sheffield UK; ^2^ Academic Urology Unit, Division of Clinical Medicine University of Sheffield Sheffield UK; ^3^ Department of Computing Sheffield Hallam University Sheffield UK; ^4^ Sheffield Institute for Translational Neuroscience University of Sheffield Sheffield UK; ^5^ Veracyte Inc South San Francisco CA USA; ^6^ Clinical Trials Research Unit University of Leeds Leeds UK; ^7^ National Heart and Lung Institute Imperial College London London UK; ^8^ Academic Oncology Unit, Division of Clinical Medicine University of Sheffield Sheffield UK; ^9^ Southampton Experimental Cancer Medicine Centre University of Southampton Southampton UK; ^10^ Department of Urology Sheffield Teaching Hospitals NHS Foundation Trust Sheffield UK

**Keywords:** urothelial carcinoma, molecular pathology, gene expression subtypes

## Abstract

The GUSTO clinical trial (Gene expression subtypes of Urothelial carcinoma: Stratified Treatment and Oncological outcomes) uses molecular subtypes to guide neoadjuvant therapies in participants with muscle‐invasive bladder cancer (MIBC). Before commencing the GUSTO trial, we needed to determine the reliability of a commercial subtyping platform (Decipher Bladder; Veracyte) when performed in an external trial laboratory as this has not been done previously. Here, we report our pre‐trial verification of the TCGA molecular subtyping model using gene expression profiling. Formalin‐fixed paraffin‐embedded tissue blocks of MIBC were used for gene expression subtyping by gene expression microarrays. Intra‐ and inter‐laboratory technical reproducibilities, together with quality control of laboratory and bioinformatics processes, were assessed. Eighteen samples underwent analysis. RNA of sufficient quality and quantity was successfully extracted from all samples. All subtypes were represented in the cohort. Each sample was subtyped twice in our laboratory and once in a separate reference laboratory. No clinically significant discordance in subtype occurred between intra‐ or inter‐laboratory replicates. Examination of sample histopathology showed variability of morphological appearances within and between subtypes. Overall, these results show that molecular subtyping by gene expression profiling is reproducible, robust and suitable for use in the GUSTO clinical trial.

## Introduction

Bladder cancer is a common malignancy that is an expensive cancer to treat [1]. In England, over 18,000 people are diagnosed with bladder cancer every year, of which approximately 25% are muscle‐invasive bladder cancers (MIBCs) [2]. MIBC has a 50% 5‐year survival rate despite radical surgery, radiotherapy and chemotherapy [3]. Cisplatin‐based neoadjuvant chemotherapy (NAC) is standard of care for MIBC and is associated with a 5% absolute increase in survival rate [4, 5]. However, individual patient responses to NAC are known to be heterogeneous. Recently, retrospective studies have shown that MIBC can be grouped into molecular subtypes based on gene expression signatures [6]. While many subtyping systems exist, a major common axis is the division of tumours into luminal/papillary and basal/squamous subtypes [6, 7, 8, 9]. Additional subtyping is possible based on the degree of tumour infiltration by immune cells and characteristics of the tumour microenvironment. Importantly, these subtypes appear to have differential sensitivity to cisplatin‐based NAC [7, 9, 10]. In retrospective cohorts, luminal tumours derive little benefit from NAC whilst basal/squamous tumours demonstrate improved survival compared with patients who received only radical cystectomy [7, 10]. This suggests that gene expression subtype‐guided care in MIBC could select patients most likely to benefit from NAC, thereby improving outcomes and reducing unnecessary treatment.

**Figure 1:**
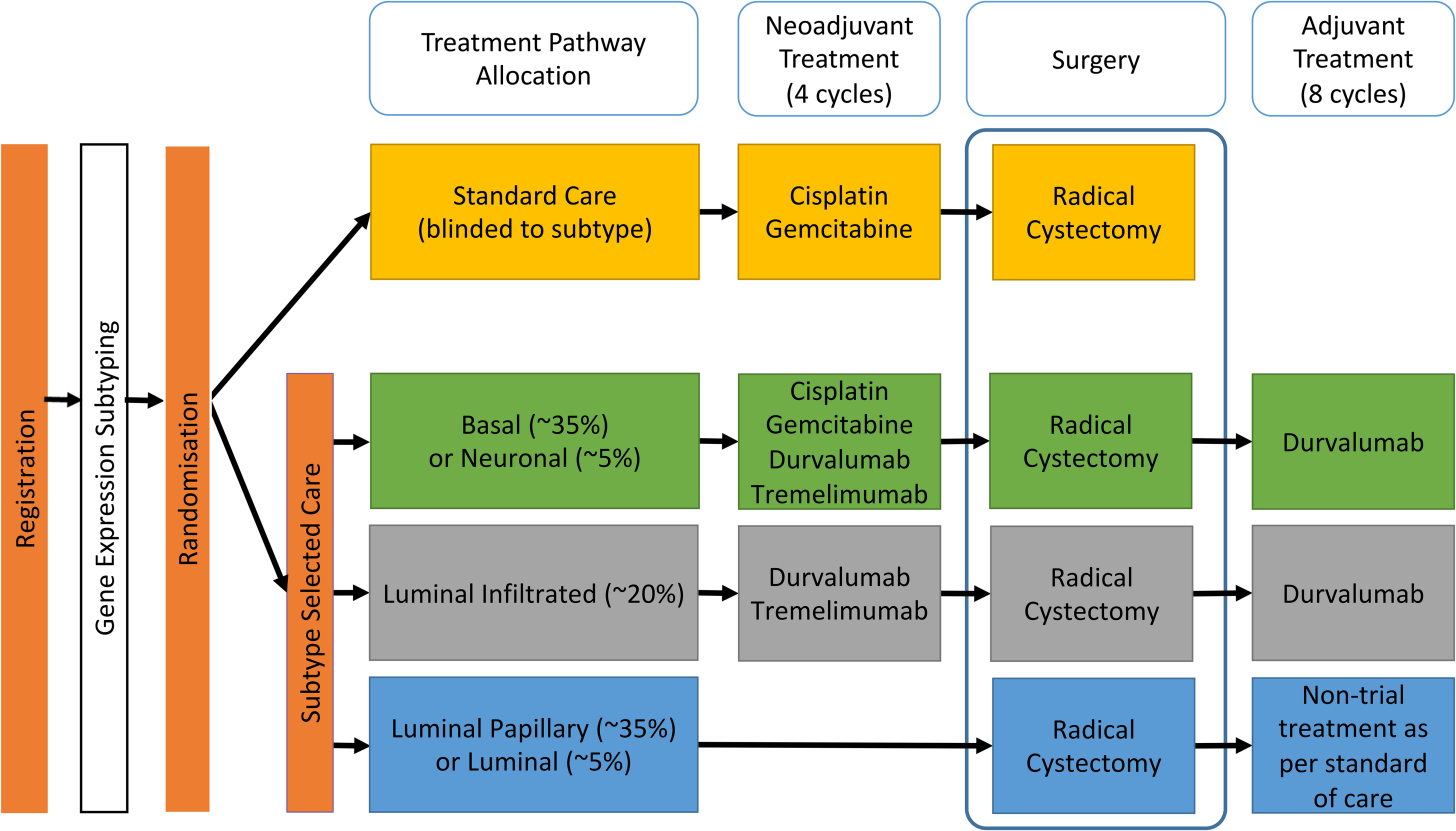
Gene expression subtypes of Urothelial carcinoma: Stratified Treatment and Oncological outcomes (GUSTO) trial schema.

GUSTO (Gene expression subtypes of Urothelial carcinoma: Stratified Treatment and Oncological outcomes) is a multicentre, prospective, open‐label, individually randomised, controlled, parallel‐group, multi‐stage phase II trial of patients with T2‐4a N0 M0 MIBC or T(any) N1 M0 MIBC who are suitable for NAC with cisplatin and gemcitabine prior to radical cystectomy (Registration: ISRCTN17378733). GUSTO will recruit 320 patients from 20 UK centres over 3 years and will assess whether there is sufficiently improved treatment activity to warrant a phase III trial. Eligible patients will be randomised to standard care comprised of neoadjuvant cisplatin and gemcitabine followed by cystectomy, or the experimental arm of gene expression subtype guided care. In the experimental arm, patients will receive treatment based on their gene expression subtype. The TCGA 2017 subtypes (luminal, luminal‐papillary, luminal‐infiltrated, basal‐squamous and neuronal) will be used [8]. Patients with luminal and luminal‐papillary tumours will proceed directly to radical cystectomy as their tumours are less likely to respond to NAC. Patients with basal/squamous and neuronal tumours will receive NAC together with systemic immunotherapy (durvalumab and tremelimumab; PD‐L1 and CTLA‐4 inhibitors, respectively). Luminal infiltrated subtype tumours may respond to PD‐L1 inhibition and patients in this group will receive systemic immunotherapy [8]. Following neoadjuvant treatment patients with basal/squamous, neuronal and luminal infiltrated subtypes will proceed to radical cystectomy (Figure 1). The primary endpoints in GUSTO are feasibility of recruitment, technical success of subtyping and pathological complete response rates.

As GUSTO uses a molecular test to guide treatment, verification of the assay in the trial laboratory is required to ensure that it is robust and reproducible. Gene expression subtyping of MIBC is available as a commercially available test (Decipher Bladder), and in this study, we used standard operating procedures for a clinical‐grade transcriptome‐wide gene expression profiling assay developed by Veracyte (San Diego, CA, USA). However, rather than using the genomic subtyping classifier (GSC; Decipher Bladder) [7, 11], we are using The Cancer Genome Atlas molecular subtyping model [8]. In GUSTO, subtype results are required as soon as possible after randomisation to avoid delays in commencing NAC or proceeding to cystectomy. The target turn‐around time is 7–10 days. Furthermore, the Decipher Bladder gene expression platform has not previously been implemented in an external laboratory in a prospective clinical trial setting. We, therefore, established the assay in an ISO15189:2012 accredited UK NHS Histopathology Laboratory to ensure we could meet the short turn‐around time required by GUSTO while maintaining the validity of the subtyping procedure. In this paper, we describe the pre‐trial verification process for the assay.

## Methods

### Samples and sample processing

Formalin‐fixed paraffin‐embedded (FFPE) tissue blocks from consecutive patients enrolled in the Investigation of Molecular Urothelial Carcinogenesis study (IMUC; South Yorkshire Research Ethics Committee (UK) reference 10/H1310/73) from 2019 to 2021 were retrieved from the histopathology archive of Sheffield Teaching Hospitals NHS Foundation Trust. Only blocks from transurethral resection of bladder tumour (TURBT) with histological evidence of MIBC were used to approximate the study population of GUSTO. One representative block was chosen for each case. Multiple 4 μm sections were cut from each FFPE block and mounted on histology slides. The first section was stained with haematoxylin and eosin (H&E) and used to guide macrodissection of viable tumour from unstained slides (supplementary material, Figure S1). Slides were marked up by the study pathologist (JG). For each case, one slide was stained with H&E after macrodissection to ensure accurate sampling. All stained slides were scanned on a Ventana DP200 (Ventana, Tuscon, AZ, USA) slide scanner at 40× (0.23 μm/pixel). Scanned slides were analysed with QuPath version 0.4.3 [12].

### RNA extraction

RNA was extracted from macro‐dissected tissue using the Qiagen FFPE RNeasy kit (Qiagen, Hilden, Germany) following manufacturer's protocols including a DNase treatment step. RNA quality and quantity were determined using a Nanophotometer (Implen, Munich, Germany) to measure RNA concentration and 260:280 and 260:230 ratios. The Agilent 2100 Bioanalyser RNA Nano kit (Agilent, Santa Clara, CA, USA) was used to assess DV200, a measure of RNA integrity and degradation expressed as the percentage of RNA fragments longer than 200 nucleotides [13]. RNA was stored at −80 °C prior to downstream analysis.

### Preparation and laboratory processing of gene expression microarrays

Reverse transcription, fragmentation, biotin labelling and hybridisation to oligonucleotide microarrays were performed as described previously [7]. In brief, 50–200 ng RNA was demodified then reverse transcribed and amplified using the Tecan Ovation FFPE WTA system (Tecan, San Mateo, CA, USA). Following quality control by a Nanophotometer, 3.5–7 μg of cDNA was fragmented and labelled with biotin using the Tecan Encore Biotin Module. Labelled cDNA was hybridised to a GeneChip Human Exon ST1.0 Array (ThermoFisher, Santa Clara, CA, USA) for 20 h at 45 °C together with positive and negative controls and alignment probes. After washes and staining, the array was imaged according to the manufacturer's instructions. The laboratory processing steps were performed for three technical replicates (two at the Sheffield laboratory and one at Veracyte) starting from RNA stored at −80 °C in aliquots. Samples were processed in different batches with different storage times between replicates to control for batch effects.

### Bioinformatics and subtype assignment

Microarray data (.CEL files) containing over 1.4 m probe sets, summarised into ~46,500 genes and non‐coding RNAs expression levels, were examined for pre‐specified quality control metrics including area under the curve (AUC) of positive versus negative control probes and percentage of probe sets on the microarray detected above background. A custom bioinformatics pipeline dedicated for individual sample analysis of trial sample data was implemented in the Genomics Resource for Intelligent Discovery (GRID) cloud‐based analytics software (Veracyte). The subtype calling procedure has been described in detail previously [7].

A custom Bash script was used to securely transfer .CEL files from the University of Sheffield secure filestore to a secure, password‐protected box.com directory by Veracyte on the GRID server; box.com is FIPS 140‐2 certified and every file is encrypted using AES 256‐bit encryption at rest and in transit. For each data upload, the Google Cloud Software Development Kit (also FIPS 140‐2‐certified) enabled login to the Veracyte filestore using a private authentication key held on the University of Sheffield secure filestore, after which an SSH connection was opened between the two servers and .CEL files transferred via SFTP. A date‐time‐stamped data transfer log was simultaneously created for each upload, giving a real‐time record of standard output and standard error streams, which detail the data transfer process and any errors resulting from the upload.

For the assignment of TCGA 2017 subtypes, each sample was assigned by a model to the nearest centroid, as defined by previous studies using the TCGA model [6, 14]. This returns correlation coefficients indicating how closely an individual sample matches each of the five subtypes. The subtype with the highest correlation coefficient was assigned to the sample.

### External verification

Following internal laboratory assay verification, remaining RNA was shipped on dry ice to be analysed in the Clinical Laboratory Improvement Amendments compliant reference laboratory which developed the assay (Veracyte). Gene expression subtyping was performed as described above.

### Immunohistochemistry

Slides were processed for immunohistochemistry using Ventana Ultra (Ventana) or Dako Omnis (Agilent) immunohistochemistry systems. Antigen retrieval, staining with primary and secondary antibodies and washes were performed as part of automated programmes optimised for each antibody. Control tissue was stained in parallel on every slide. Details for each antibody are given in supplementary material, Table S1.

### Data analysis and statistics

Data were analysed and figures were created using GraphPad Prism 9 version 9.3.1 and R version 4.0.3 [15]. Details of statistical tests are given in the relevant figure legends.

## Results

### Cohort details

The cohort comprised 18 patients (14 male) with a median age at diagnosis of 76 (range, 53–93). All cases had histological evidence of detrusor muscle invasion and were high grade (WHO/ISUP 2004) at TURBT. Carcinoma *in situ* (CIS) was present in 12 cases. FFPE blocks were stored at room temperature for a median of 55 months (range, 44–96) prior to RNA extraction.

### Quality and quantity of RNA and cDNA

For practical implementation of block selection and RNA macrodissection in GUSTO, it is important to determine how many unstained slides are required to achieve sufficient RNA yields. We, therefore, asked if RNA concentration correlated with the area of tumour dissection. We tested three methods for estimating the tumour area. First, we used a simple method of measuring each tumour area in two perpendicular directions on the glass slide, multiplying these two numbers and summing the combined areas if more than one area was measured. This gives an approximate tumour area. Second, we used the scanned whole slide image and QuPath to digitally measure the marked‐up area. Third, we measured the total number of cells in the digitally marked‐up area.

**Figure 2:**
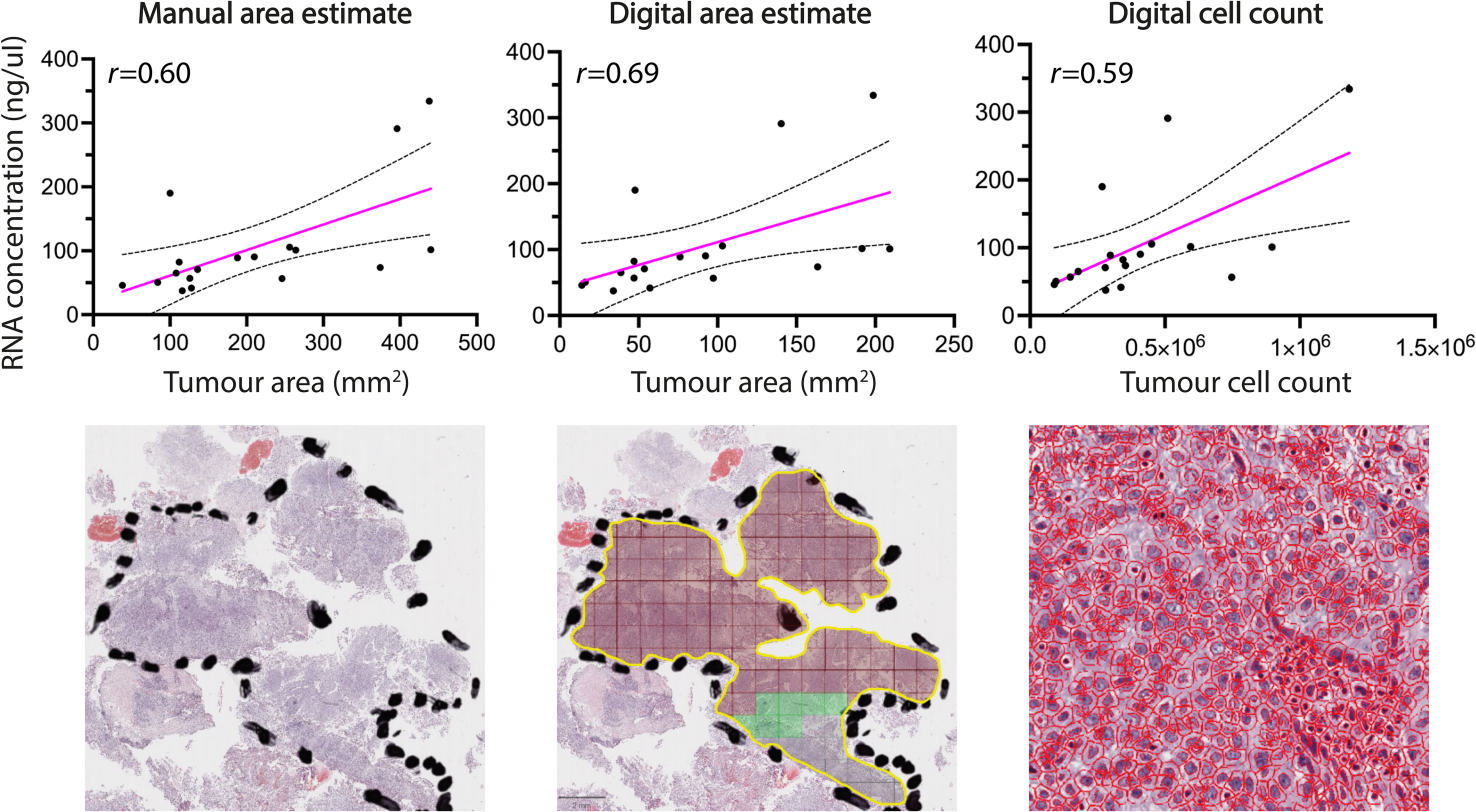
Manual markup and macrodissection yields sufficient RNA from FFPE TURBT samples. Method of area measurement or cell count versus RNA concentration is shown. The Spearman correlation coefficient (*r*) for each comparison is indicated on each graph. The pink straight line shows the line of best fit derived from least squares linear regression. The dotted lines indicate 95% confidence intervals. Representative H&E images of each method are shown underneath the corresponding graphs.

Sufficient RNA was extracted from all TURBT blocks. The median RNA concentration was 78.1 ng/μL (range, 37.5–334.1 ng/μL). The 260/280 ratio was above 1.7 (range, 1.7–2.2) for all samples indicating RNA of high purity. We found similar correlation coefficients between each method of measuring tumour area or cellularity and RNA yields (Figure 2). All blocks yielded more RNA than the minimum input (20 ng/μL) required for reverse transcription and amplification and 16/18 samples had an RNA yield of >100 ng/μL. The median percentage of RNA molecules longer than 200 nucleotides (DV200) was 45.5% indicating moderate RNA degradation (supplementary material, Figure S2A).

Following reverse transcription and amplification, we measured the quantity and 260:280 ratio of the resulting cDNA in technical replicates. The mean cDNA concentrations were 241.2 and 237.8 ng/μL for replicate one and two, respectively. The difference between the replicates was not statistically significant (supplementary material, Figure S2B). The 260:280 ratio was >1.9 for all samples. These values exceeded the manufacturer's lower limit for use in a microarray in all samples across technical replicates. A nuclease‐free water control was processed in parallel with every sample, which generated lower cDNA quantities indicating no significant contamination during the reverse transcription and amplification steps. We observed low 260:230 ratios for RNA samples (supplementary material, Figure S2C) but this did not affect the reverse transcription step as all cDNA 260:230 ratios were >2.2 (supplementary material, Figure S2D).

### Quality control of array data and concordance of gene expression subtyping

**Figure 3:**
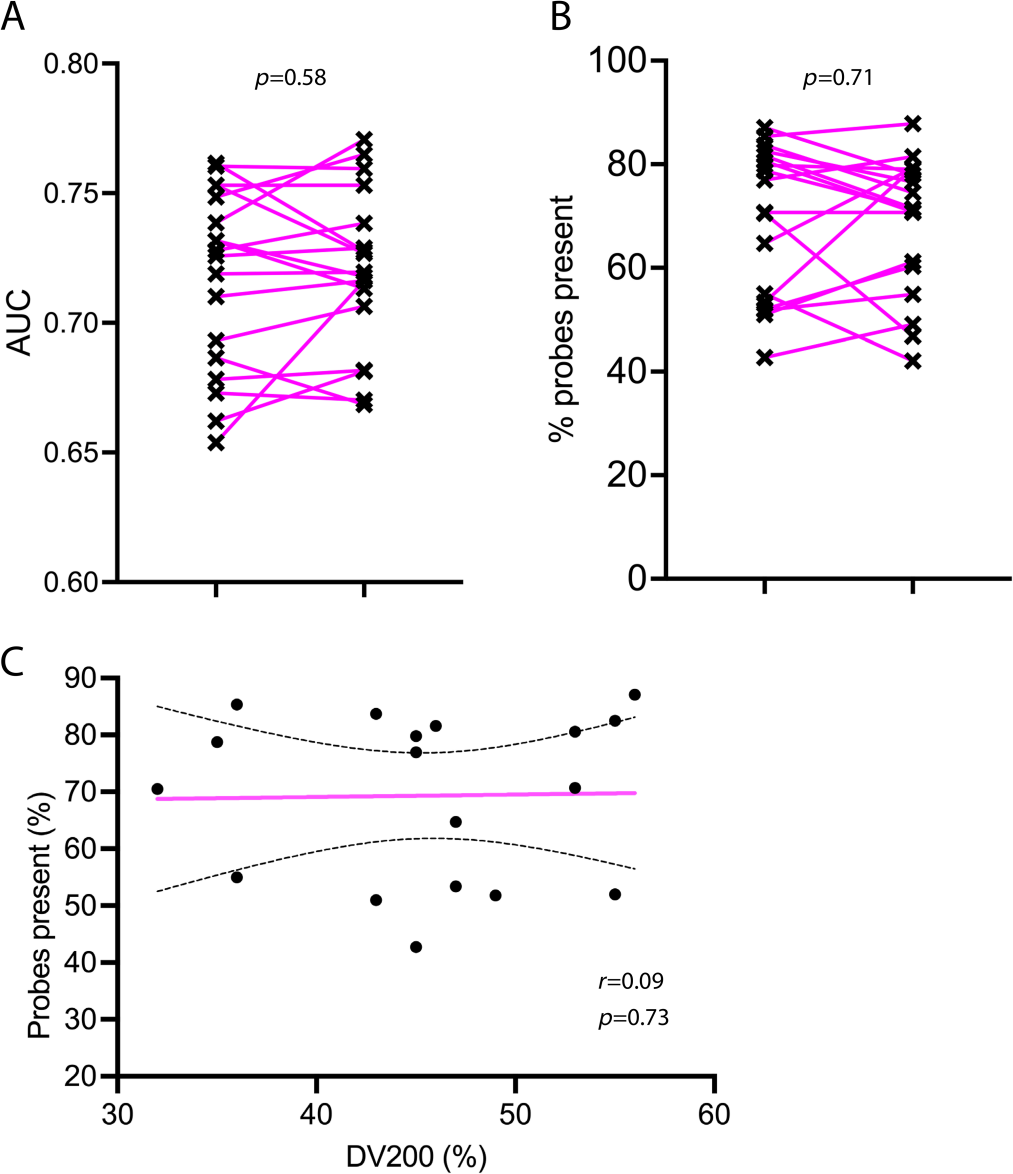
Quality control metrics of gene expression microarrays. (A) Area under the curve (AUC) of internal positive controls (exonic sequences) versus internal negative controls (intronic sequences). (B) Percentages of probes present. This metric indicates probe signals detected above the background for all genes in the microarray. Both metrics are paired comparisons between technical replicates in the Sheffield laboratory. Pink lines indicate pairs of observations. The p‐values were derived from two‐tailed paired student *t* test. (C) DV200 versus percentage probes present. *r* is Spearman's correlation coefficient. The pink straight line shows the line of best fit derived from least squares linear regression. The dotted lines indicate 95% confidence intervals.

The positive versus negative control AUC of internal control probe sets was >0.65 for all samples, demonstrating adequate signal‐to‐noise ratio for the detection of exonic versus intronic sequences above background. No significant difference was observed in AUC between technical replicates performed in the same laboratory (Figure 3A). The percentage of probes present (PPP) above the background ranged from 40% to 85% and did not differ significantly between technical replicates (Figure 3B). PPP is a quality control measure for gene expression microarrays. Suboptimal laboratory procedures would produce poor hybridisation and, therefore, a low PPP. We also observed no correlation between RNA 260:230 ratios and the PPP for each sample (Figure 3C). This indicates that any carryover or contamination from the RNA extraction process did not affect the downstream assay steps of reverse transcription, fragmentation and labelling and probe hybridisation.

**Figure 4:**
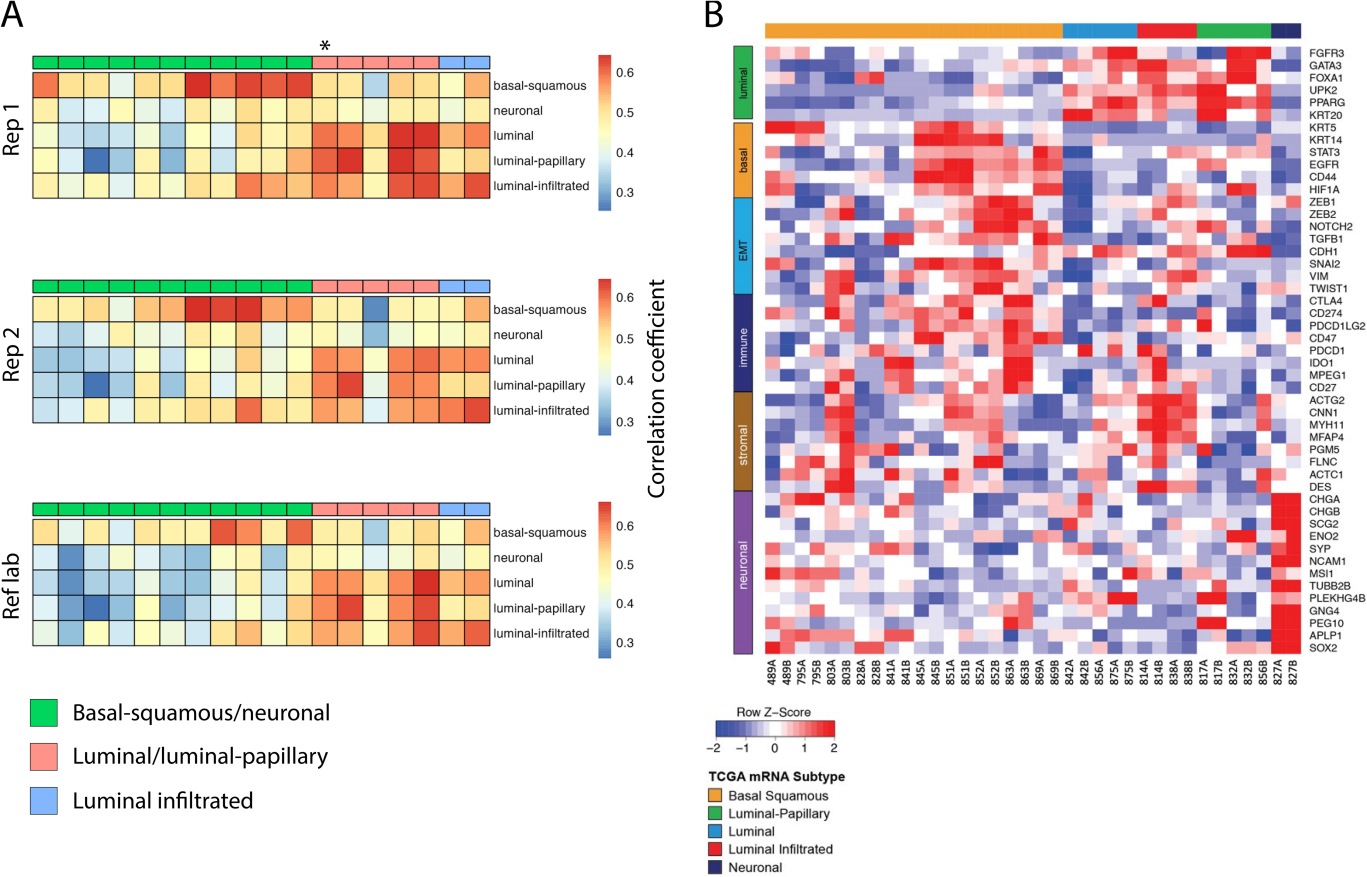
Gene expression subtyping shows intra‐ and inter‐laboratory concordance. (A) Correlation between subtypes and technical replicates. Each column is an individual sample. Each subtype is represented by an idealised centroid composed of expression values for genes that best discriminate that subtype from others. Normalised expression values from a sample are compared with each centroid and the subtype with the highest correlation coefficient being most similar and assigned to the sample. Sample annotations have been grouped to reflect the three different treatment arms within GUSTO. The asterisk indicates one case with a discrepant subtype call of luminal versus luminal papillary between technical replicates 1 and 2 (rep 1 and rep 2, respectively. Ref lab is the reference laboratory result). (B) Heatmap of normalised expression values indicating genes that contribute to each subtype.

Fifty‐four subtypes were returned across the three technical replicates. The same subtype was returned in 17/18 samples in all three replicates. In one sample, there was a luminal papillary versus luminal discrepancy in the two replicates subtyped by the Sheffield laboratory. The external laboratory verification returned this subtype as luminal papillary (Figure 4). Similar intra‐ and inter‐laboratory reproducibility rates were observed using other subtyping classifiers such as the Consensus [14] and GSC (Decipher Bladder) [7] models (data not shown).

### Morphological and molecular heterogeneity

**Figure 5:**
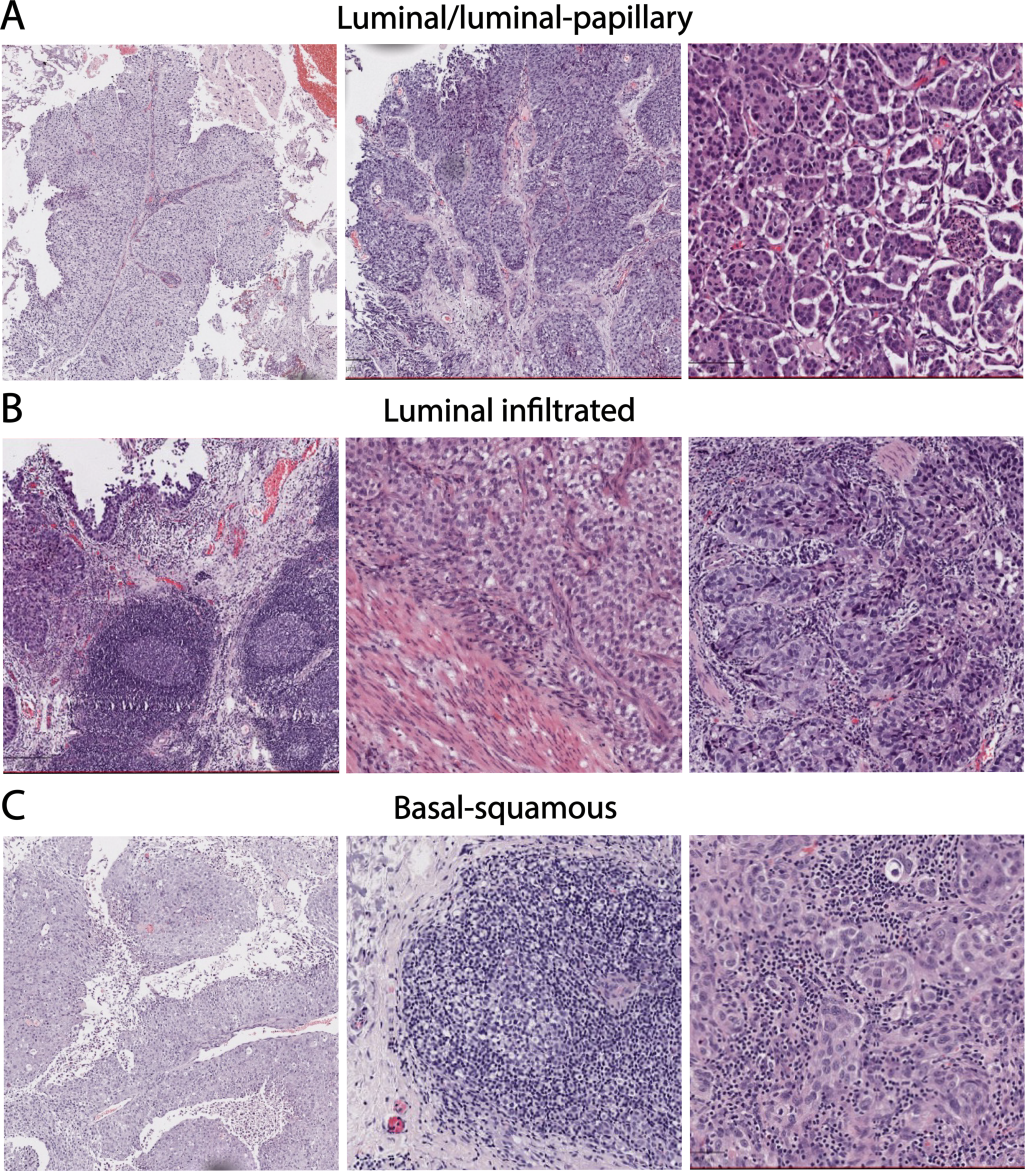
Representative examples of morphological features across subtypes. (A) Luminal/luminal papillary subtype tumours with papillary (left), papillary and infiltrating (middle) and micropapillary (right) morphology. (B) Luminal infiltrated subtype tumours with tertiary lymphoid structures (left), infiltrating urothelial cell carcinoma adjacent to detrusor muscle (middle) and prominent tumour infiltrating lymphocytes (right). (C) Basal subtype tumours with papillary morphology (left), tertiary lymphoid structures (middle) and squamous morphology with prominent tumour infiltrating lymphocytes (right).

**Figure 6:**
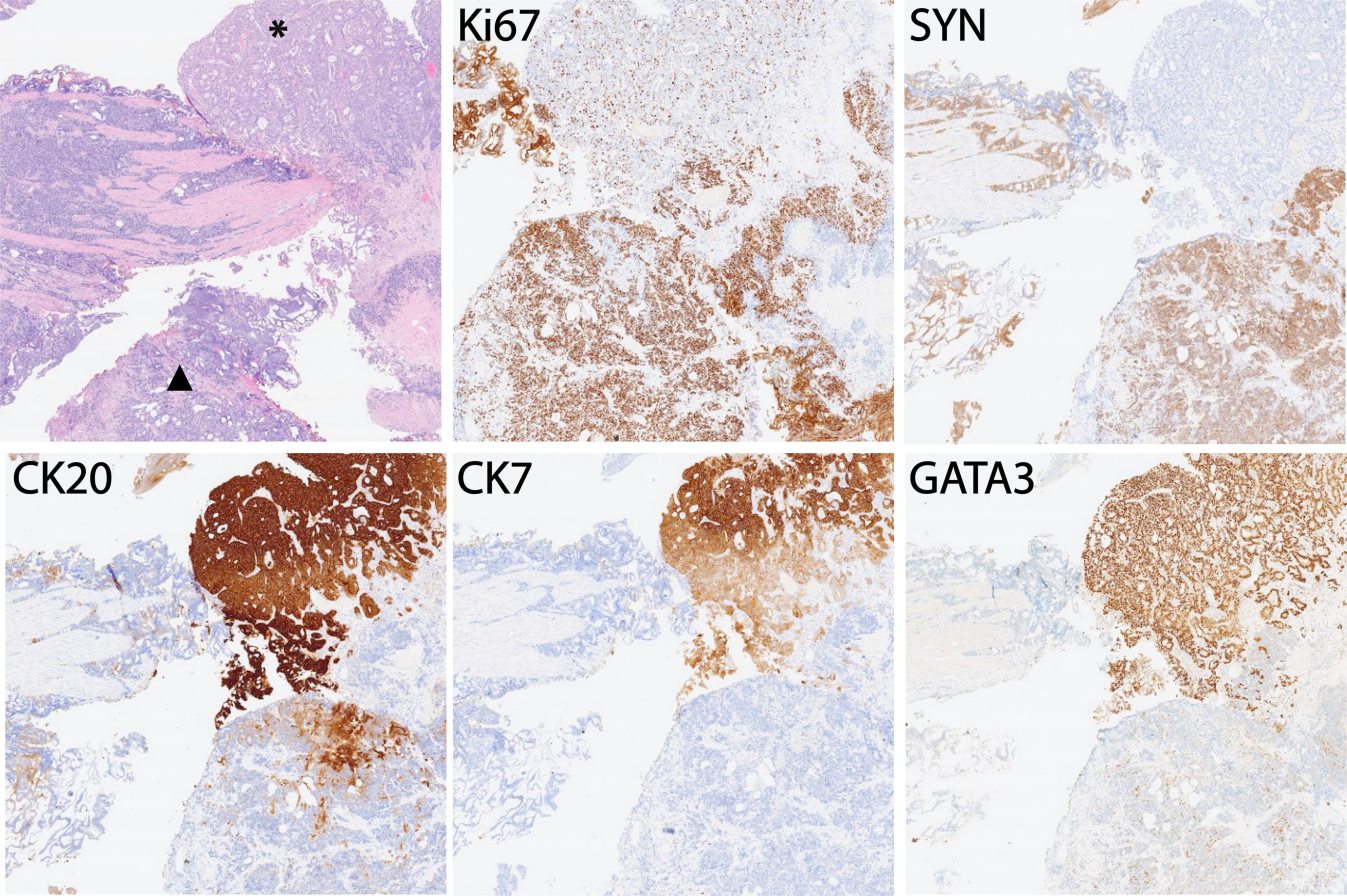
Neuronal subtype tumour with multiple morphological appearances. The adjacent glandular (asterisk) and high‐grade neuroendocrine (black triangle) components are shown together with immunohistochemistry for KI67, synaptophysin (SYN), CK20, CK7 and GATA3. Supplementary material, Figure S3, shows the majority of high‐grade neuroendocrine component.

All TURBT slides were reviewed by the study pathologist (JG) and areas of papillary, non‐papillary and divergent differentiation were recorded. In addition, the formation of tertiary lymphoid structures (TLSs) with germinal centres was assessed as this has recently been described as a putative biomarker for response to PD‐L1 inhibitors [16, 17]. Twelve of eighteen cases contained more than one morphological pattern (Table 1). Of the 10 basal‐squamous cases, seven had papillary morphology at least focally. Similar morphology was present more extensively in the two cases with luminal‐papillary subtype. In addition, one of these cases contained micropapillary architecture. TLSs were present in three cases (Figure 5). The cohort contained one neuronal subtype tumour. This case had poorly differentiated morphology with a background of CIS. The poorly differentiated areas expressed CD56 and synaptophysin without GATA3 expression. The KI67 labelling index in these areas was >90%. This tumour also contained small foci of glandular differentiation and squamous morphology, both of which lacked expression of neuroendocrine markers but retained GATA3 expression indicating urothelial differentiation in this context [18, 19] (Figure 6). However, the majority of the tumour had poorly differentiated/neuroendocrine morphology with concordant expression of synaptophysin and high KI67 labelling index (supplementary material, Figure S3). Taken together, these results suggest that morphological features alone may not correlate well with molecular subtypes.

**Table 1:** Morphological features of tumours from TURBT stratified by gene expression subtype

Morphology	Papillary	Squamous	High grade invasive	Poorly differentiated	Glandular	CIS	TLS
Subtype							
Basal (*n* = 10)	7	2	10	1	0	7	2
Neuronal (*n* = 1)	0	1	0	1	1	1	0
Luminal/luminal papillary (*n* = 4)	2	1	3	1	0	2	0
Luminal infiltrated (*n* = 2)	0	0	2	0	0	2	1

CIS, carcinoma *in‐situ*; TLS, tertiary lymphoid structures.

## Discussion

GUSTO is the first randomised clinical trial to test gene expression subtyping for neoadjuvant treatment decisions in bladder cancer. Here, we have shown that subtyping using this gene expression microarray platform and TCGA subtypes is robust and reproducible. RNA is regarded as an unstable molecule prone to degradation and subsequent assay variability, particularly in the context of FFPE tissue [20]. We successfully extracted RNA of sufficiently high quality and quantity for multiple technical replicates of gene expression subtyping in 18 FFPE bladder cancer samples. During this verification study, we found that manual markup and overlay of the annotated slide with unstained slides provided the easiest method of macrodissection (supplementary material, Figure S2). This method provided adequate RNA yield for all samples from two unstained sections across a range of tumour areas. From this observation, we have selected a minimum manually measured tumour area of 50 mm^2^ for use in GUSTO. We have also shown manual markup and measurement is an easy technique that can be implemented in any laboratory. While slide scanning and digital pathology are used by many departments, including our own [21, 22], there is variation in use across the UK [23]. As GUSTO is recruiting from 20 UK centres with potentially varying digital pathology access, manual markup will be used in GUSTO. However, in other settings using very small samples or where remote pathologist input is required, digital measurement linked to automated macrodissection may be useful [24].

No samples failed quality control at any stage of the subtyping process. Despite two freeze‐thaw cycles, storage times of up to 12 months, shipping of RNA samples and processing by two separate laboratories, similar subtype results were returned for each sample. We demonstrate a median DV200 of 45.5%, which is comparable with other studies using RNA from FFPE tissue [25]. Despite this moderate level of RNA degradation, all samples were successfully subtyped indicating that the assay was robust to RNA degradation and appropriate for use in FFPE tissue. This is likely due to the short probe lengths (25 nucleotides) in the Affymetrix array [26] being able to hybridise to RNA less than 200 nucleotides in length and is a potential advantage of microarray platforms over RNA‐seq which typically requires longer RNA molecules. We also found that extracted RNA had low 260:230 ratios. Carry‐over of guanidinium chloride is the commonest cause for a low 260:230 ratio. However, spike‐in experiments have previously demonstrated that this does not affect downstream experimental procedures such as reverse transcription and library preparation [27]. Our experience mirrors this as we found all samples achieved adequate cDNA yields and purity. Furthermore, all samples generated valid array data.

One discrepant result occurred in the verification process. Two of three technical replicates returned a subtype of luminal, whereas the remaining replicate returned a subtype of luminal‐papillary. These two subtypes are more similar to each other than pairwise comparison of any other of the subtypes. It follows that they would be the most susceptible to subtype ‘swapping’ owing to technical variations in sample processing. Importantly, in GUSTO, luminal and luminal papillary tumours receive the same treatment in the subtype guided care arm of the trial. Therefore, this discrepancy would not be clinically significant in the trial.

The FOCUS4 trial laboratory has described pre‐ and within‐trial validation [28, 29]. These studies highlighted the importance of performing inter‐laboratory reproducibility assessments, including resolving discrepant results between laboratories. FOCUS4 used biomarkers based on DNA sequencing and immunohistochemistry, and the trial data showed that these are robust and reliable tests. Both DNA and protein have good stability compared with RNA stored in FFPE blocks. Furthermore, these biomarkers were single gene tests measuring either the presence/absence of multiple mutations or protein expression. In contrast, the RNA‐based gene expression subtype assay used in GUSTO is based on gene expression microarray technology. It measures the expression of more than 46,000 genes overall and uses about 200 genes to generate subtypes. The laboratory procedures and data processing/bioinformatics required for single‐sample classification in the Decipher bladder assay are more complex than single gene mutation testing or immunohistochemical analysis of protein expression. Despite this increased complexity of both laboratory and bioinformatics processes, subtyping in our verification cohort showed good intra‐ and inter‐laboratory reproducibility and was robust to variability encountered in real‐world laboratory practice.

Finally, we have shown morphological heterogeneity within and between gene expression subtypes. This indicates the value of performing subtyping to select for NAC, as this molecular test provides information beyond histomorphological appearances. A limitation of our study is the small sample size. Any verification study is a balance between capturing biological variability across a population, demonstrating technical reproducibility and cost. Our cohort of 18 FFPE TURBT samples represented every gene expression subtype that will be used in the GUSTO trial and a variety of morphological appearances. By performing technical replicates and external laboratory verification, we have shown that the assay is robust and suitable for use in GUSTO. Furthermore, within the prospective phase of the GUSTO clinical trial, there will be ongoing assay verification to ensure the quality of the assay is maintained.

## Author contributions statement

JG, JD and PRH performed experiments. LAQ, EAG, ED, YL, XZ and DW undertook bioinformatic analysis. JG, JD, EAG and JWFC carried out data analysis. All authors designed the research and drafted the manuscript or revised it critically for important intellectual content.

## Supporting information


**Figure S1.** Macrodissection of unstained sections
**Figure S2.** Additional quality control measures of RNA extracted from FFPE tissue and corresponding cDNA following reverse transcription
**Figure S3.** H&E and IHC from areas of the tumour classified as neuroendocrine by gene expression profiling
**Table S1.** Antibodies used for immunohistochemistry

## Data Availability

Raw .CEL gene expression microarray files are available through the Gene Expression Omnibus (https://www.ncbi.nlm.nih.gov/geo/), accession number GSE253531.
