# Supplementary material for: Verification of molecular subtyping of bladder cancer in the GUSTO clinical trial
Source: J Pathol Clin Res. 2024 Feb 1;10(2):e12363. doi: 10.1002/2056-4538.12363 (PMC10835016; doi:10.1002/2056-4538.12363)
Supplement: Supplementary file 1 — Figure S1. Macrodissection of unstained sections Figure S2. Additional quality control measures of RNA extracted from FFPE tissue and corresponding cDNA following reverse transcription Figure S3. H&E and IHC from areas of the tumour classified as neuroendocrine by gene expression profiling Table S1. Antibodies used for immunohistochemistry [file CJP2-10-e12363-s001.pdf]

Verification of gene expression subtyping of bladder cancer in the GUSTO clinical trial

J Griffin, J Down *et al.*, *J Pathol Clin Res*, https://doi.org/10.1002/2056-4538.12363

Supplementary Figures S1–S3
Supplementary Table S1

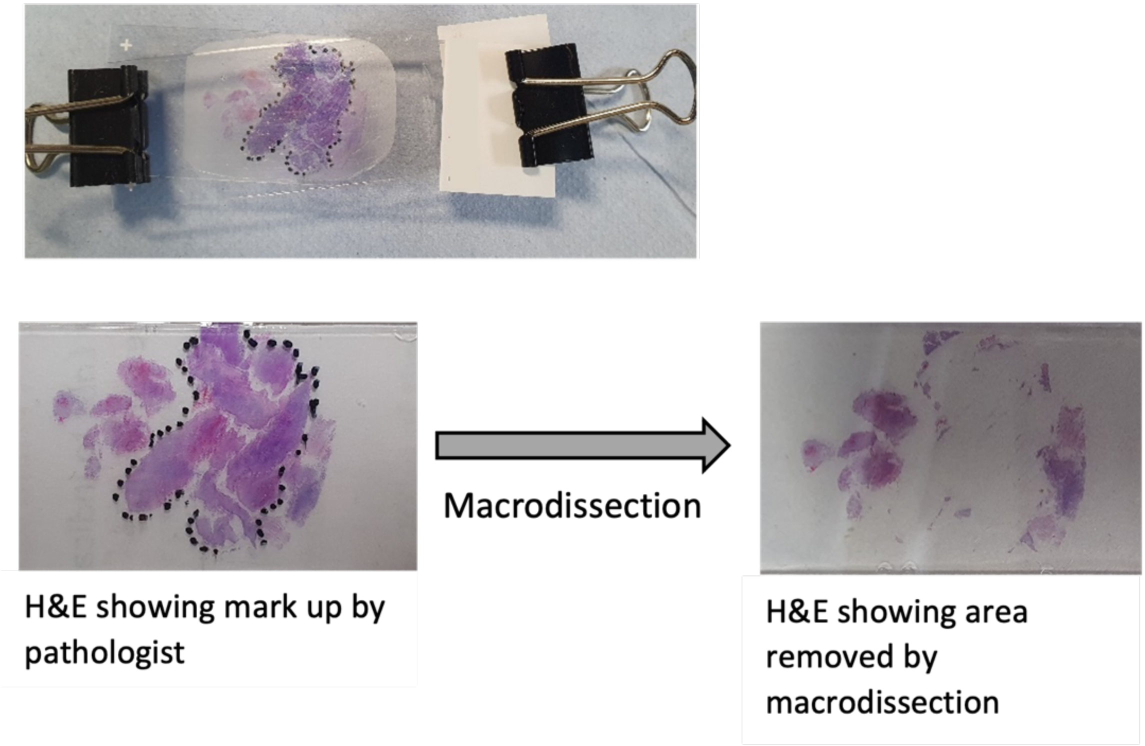

Figure S1. Macrodissection of unstained sections. Upper: Manual mark up and overlay of H&E stained slide with unstained sections. Lower: H&E of sections adjacent to unstained sections showing successful macrodissection of marked area.

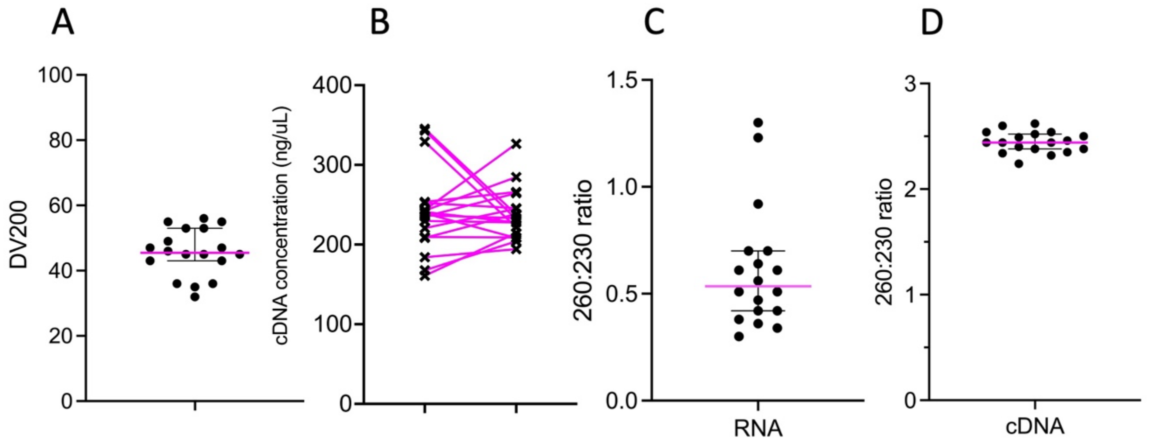

Figure S2. Additional quality control measures of RNA extracted from FFPE tissue and corresponding cDNA following reverse transcription. (A) DV200 expressed as the percentage of RNA molecules >200 nucleotides in length. (B) cDNA concentrations of two technical replicates processed in the Sheffield laboratory. (C) 260/230 ratio of RNA samples used in the verification study. (D) 260/230 ratios of cDNA made from the RNA samples in A and C.

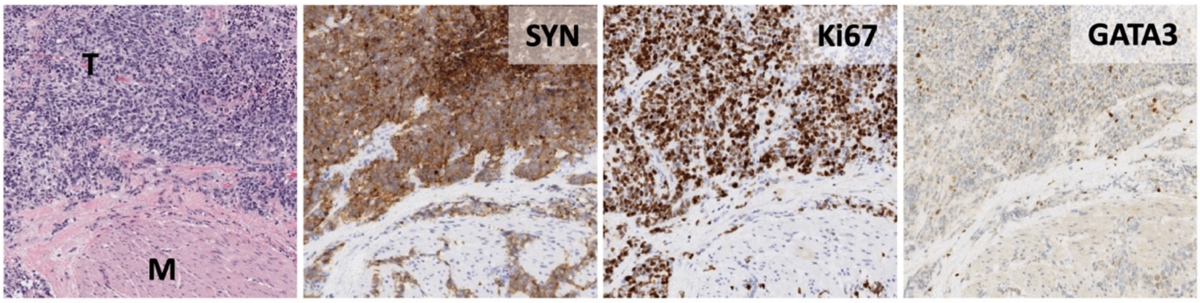

Figure S3 (relates to figure 6 in the main text). H&E and IHC from areas of the tumour classified as neuroendocrine by gene expression profiling. The majority of the tumour was a poorly differentiated malignancy (tumour; T) widely invading detrusor muscle (M). The tumour cells expressed synaptophysin in keeping with neuroendocrine differentiation. KI67 labelling index was >95%. GATA3 IHC labelled background lymphocytes only in areas of poorly differentiated neuroendocrine carcinoma.

Table S1. Antibodies used for immunohistochemistry

**Antibody**	**Manufacturer**	**Product number**	**Species**	**Dilution**	**System**	**Antigen retrieval**
Ki67	Dako	GA626	Mouse	Purchased RTU	Dako Omnis	Low pH, 97 °C for 30 mins
GATA3	Cell Marque	760 – 4897	Mouse	Purchased RTU	Ventana	95 °C for 56 mins
CK7	Dako	GA619	Mouse	Purchased RTU	Dako Omnis	Low pH, 97 °C for 30 mins
CK20	Dako	GA777	Mouse	Purchased RTU	Dako Omnis	Low pH, 97 °C for 30 mins
Synaptophysin	Dako	M7315	Mouse	1:50	Dako Omnis	Low pH, 97 °C for 30 mins

RTU: Ready to use
